# At the junctures of healthcare: a qualitative study of primary and specialist service use by Polish migrants in England

**DOI:** 10.1186/s12913-022-08666-z

**Published:** 2022-11-03

**Authors:** Giuseppe Troccoli, Chris Moreh, Derek McGhee, Athina Vlachantoni

**Affiliations:** 1 Centre for Research on Ageing and Centre for Population Change, University of Southampton, Southampton, UK; 2 School of Geography, Politics and Sociology, Newcastle University, Newcastle upon Tyne, UK; 3 Faculty of Social Sciences, University of Stirling, Stirling, UK

**Keywords:** Transnational healthcare, Migration, Primary care, General Practice, Specialist care, United Kingdom, Poland, NHS/National Health Service, Culture, Private healthcare

## Abstract

**Background:**

Polish people are the biggest migrant group in the UK and the scholarship shows that they are attentive to their healthcare needs and seek to fulfil them by using various services both within and outside the British public healthcare system. This article explores the role of junctures *within* healthcare systems in the connections migrants realize *between* healthcare systems and sectors. The article argues that in a transnational context, migrants enact these junctures by joining different levels of care within the same sector, between sectors and across national borders. In particular, the article explores how Polish migrants’ healthcare seeking practices within and beyond national borders are enacted given the features, availability and relationship between primary and specialist care for how they are articulated between private and public sectors.

**Methods:**

This article is based on the second phase of a mixed-methods study on how Polish people in the UK manage their health transnationally. The participants were purposefully sampled from survey respondents (first phase) who identified as having a long-term health condition or caring in a non-professional capacity for someone who is chronically ill. Thirty-two semi-structured audio-call interviews were conducted with Polish migrants living in England between June and August 2020. Transcripts were analysed by applying thematic coding.

**Results:**

Key findings include a mix of dissatisfaction and satisfaction with primary care and general satisfaction with specialist care. Coping strategies consisting in reaching specialist private healthcare provided a way to access specialist care at all or additionally, or to partially complement primary care. When Polish private specialists are preferred, this is due to participants’ availability of time and financial resources, and to the specialists’ capacity to fulfil needs unmet within the public healthcare sector in the UK.

**Conclusion:**

Polish migrants join with their practices systems which are not integrated, and their access is limited by the constraints implied in accessing paid services in Poland. This shapes transnational healthcare practices as relating mostly to routine and ad-hoc access to healthcare. These practices impact not only the wellbeing of migrants and the development of the private market but also the public health provision of services.

## Introduction

So, I think your research will show that access to NHS is easy and largely free of charge, but if Polish people have to pay, they prefer to go to Poland. (Jakub)

I feel more comfortable speaking to Polish doctors; they will spend half an hour explaining to me about the nature of the problem and its cause; they’ll explain the mechanisms behind the problem; they’ll tell me everything. (Dorota)

I think Polish people who have a tendency to want to be taken care of… But you need to come up with an initiative yourself and take what you’re being offered, and there is a lot on offer - I am very pleased with it - you just need to go and take it… (Karolina).

Jakub, Dorota, and Karolina are part of the 738,000 Polish nationals currently living in the UK [1]. Their words seem to be contradictory statements about British and Polish healthcare. They point to ease and discontent with healthcare services, as well as crude calculation of economic viability and cultural preference in healthcare seeking. Taken separately, these words reflect a range of views and practices which are usually described by considering barriers and facilitators to migrants’ access to healthcare in their country of residence and back in their country of origin. Taken together, they suggest a variety of behaviours which result from the interactions of singular factors such as the quality of healthcare or one’s satisfaction with healthcare services, which in turn explain individuals’ preferences and behaviours. In this article, we make a unique contribution to existing scholarship by exploring these apparent contradictions and situating them within a transnational framework attentive to connections and movements between borders, as well as past and ongoing experiences, while emphasizing migrants’ use of service in their country of residence. To understand migrants’ healthcare, we situate them within shifts between borders, institutions, and markets. Migrants enact articulations within and between public and private providers when attending to their needs according to the availability and organization of healthcare services. Polish migrants to the UK like Jakub, Dorota and Karolina are attentive to their healthcare needs and seek to fulfil them by using a variety of services both within and outside the British public healthcare system. To understand these practices, we draw from the qualitative phase of a mixed-methods project consisting of thirty-two semi-structured interviews with Polish migrants living in England.

## Background

The global literature on the topic shows that migrants manage their health through a variety of health-seeking behaviours which reflect their resilience and creativity in tactically combining resources at the local and transnational levels [2, 3]. Approaches that consider migrants returning to their country of origin as part of a wider global flow of cross-border healthcare describe patient mobility in terms of their motivations and available funding, exploring the factors which motivate individual patients to seek healthcare abroad [4, 5]. When these practices are understood as instances of medical tourism the literature highlights a series of factors involved in access to healthcare services in the migrant’s country of origin, including the medical culture, time availability, communication, dissatisfaction with the healthcare system, status of healthcare insurance, quality of healthcare, seeking a second opinion, and affordability [6]. These factors represent recurrent and pivotal elements of individuals’ medical travels. However, grouping them as push and pull or motivational factors could reduce the migrants’ experiences to those of consumers, thereby hiding the complexities and interplay behind each of these factors. Indeed, critics of medical tourism have highlighted that this concept masks relationality and connections within which health seeking takes place [7], and that it does not adequately capture the breadth of these practices [4, 8], that it implies the experience of pleasure and conceals the economic efforts by patients [9], and finally that it is inadequate to describe the complexity of migrant flows in the case of patients’ movements from the UK to Poland [10].

A different take consists in exploring migrant’s health coping strategies within the wider transnational paradigm and placing them within various stratification systems in their use of transnational ties, which allow (to a certain extent) to both overcome local barriers and augment their possibilities in terms of accessing different types of care [11]. The use of healthcare services by migrants is shaped as much by their past experiences [12] as by their ongoing engagement with services given their changed economic circumstances [13, 14]. Crude cost calculations and a preference for one’s home country’s medical culture need to be situated within migrants’ status transformations within which their medical returns occur [14]. Transnational healthcare practices are embedded in class inequalities in both home and host countries, and through them, migrants participate in the privatization of healthcare services across borders [15, 16] and in changing medical practices [17]. Further, transnational perspectives reveal that healthcare practices are not confined to returning home for healthcare but are often multiple, and comprise movements in different directions between countries, sectors and providers [18, 19]. In this article, we take a transnational healthcare perspective that considers migrants’ changing positionalities and ongoing engagements across borders within the social and economic contexts in which they operate. The added value of our contribution consists in exploring the role of junctures *within* healthcare systems in the connections migrants realize *between* healthcare systems and sectors. This entails keeping the analytical relevance of shifts and transformations as they are enacted between borders and in time, while recognizing that migrants pursue healthcare vis a vis the systemic features of healthcare systems in which they are embedded and to which they gain access. This allows us to appreciate how the understanding of care, and the healthcare needs and seeking practices emerge differentially in individuals’ interactions with the healthcare systems. In turn, such appreciation expands the study of transnational healthcare by adding analytical specificity to the scrutiny of the emergence of migrants’ healthcare practices across borders. We argue that in a transnational context, migrants enact these junctures by joining different levels of care within the same sector, between public and private sectors and across national borders. In particular, we explore how Polish migrants’ healthcare seeking practices within and beyond national borders are enacted given the features, availability and relationship between primary and specialist care for how they are articulated between private and public sectors.

### Polish migrants’ healthcare practices and transnational healthcare

Studies which documented the experiences and attitudes of Polish migrants in the UK are consistent in highlighting the elements of primary care which are commonly object of critique. While having a variety of experiences within the British healthcare system and unproblematic access to it, a common trend in the opinions of the Polish women living in London of Main’s [20] ethnography was their critique of what they named the ‘English approach’ (Ibid. p57, our translation): the medical professionals’ reluctance to refer to specialists, dismissal of symptoms, prescription of paracetamol and recommendation to rest. In research conducted by Osipovič [21], London-resident Polish interviewees were also dissatisfied with waiting times and had to persevere to be referred to specialist consultations. A study based on focus groups and interviews in a Northern England town with Eastern European migrants mainly from Poland [22] found high frustration with GPs (General Practitioners), who were perceived as being dismissive, short on time, and at times difficult to reach at a short notice. Participants reported that GPs did not properly examine patients, dismissed symptoms, recommended rest and prescribed paracetamol instead of therapies, and did not refer them to specialist care. A minority of respondents, who lived in the UK for over ten years, were proficient in English and were University educated, appreciated the UK approach promoting self-care, and criticized the overreliance on medications of some of their compatriots. Two other qualitative studies involving Polish and Romanian participants, in both cases with a majority of the former nationality, living across Scotland [23] and in different areas of England [24] found a similar level of disappointment about waiting times, referrals and treatment approaches (see also [25]). From this literature, it emerges that the dissatisfaction of Polish people in the UK towards British public healthcare is chiefly with primary care and its gatekeeping function towards specialized care.

By contrast, the experience of accessing specialist care has received less and more scattered scrutiny in the literature. Some research in England and Scotland has been devoted to the evaluation of uptake of specific public health measures in defined localities, including preventive screening [26, 27] and vaccination programmes [24, 28–30]. Studies considering Polish women’s experiences with maternity care found both dissatisfaction and appreciation for the structural features and approaches to care found in the UK when compared with Poland [20, 31, 32].

Another aspect of the literature considering the health-seeking of Polish people in the UK is their use of private services, most commonly accessed back in Poland usually combined with family visits enabled by relatively cheap flights and ease of travel [20, 21, 23, 26, 27, 33]. The magnitude of the phenomenon is shown by the increase in air travel from the UK to Poland primarily for healthcare reasons by the growing Polish population resident in the UK [10]. Polish people also access clinics staffed by Polish professionals offering health services targeting this migrant population [20, 21]. To a lesser extent, Polish people also access private healthcare services in the UK from other providers [18]. Limited understanding of the functioning and entitlement to public healthcare has been found in earlier research to be associated with the use of services in Poland and of private Polish clinics in the case of recently arrived migrants or undocumented workers [21, 23, 33]. Successive research has found that these services are accessed even amongst migrants aware of their entitlements and the functioning of the medical system [20, 22].

Services offered by Polish professionals have been found to appear trustworthy, because of the familiarity of medical professionals’ approach to patients and medical practices or because such services were already known before migrating or accessed through networks [21, 22, 25]. Consistently, accessing private medical care, usually in Poland and mostly paid out-of-pocket, has been found as a way to bypass waiting times and limited access to specialists [20, 21, 23, 24, 33]. Main [20] found that for her participants the most important reason to seek health in a country different from the one of residence was the level of satisfaction with local healthcare, in particular the accessibility to specialist care. The phenomenon of healthcare access in the country of origin has been documented among Polish people in other countries in the European Economic Area [5, 34–37], as well as among Europeans [38] and other migrants in the UK [39–41].

In this article, we develop the findings that point to the difference in approaches to specialist and primary care to show how these are central in both the understanding of healthcare services received and in shaping the kind of services that are accessed nationally and transnationally. We take the ways in which the juncture between primary and specialized care is articulated from the migrants’ perspective in their interactions with the healthcare system in the UK, in Poland, and between the two countries. Thus, we reframe the migrants’ ongoing and past healthcare seeking practices within changing circumstances in terms of their enactment of this particular juncture.

### British and Polish healthcare systems

The United Kingdom has a healthcare system that is mostly free at the point of access for legal residents. Dental care and ophthalmic services, and in England outpatient prescribed drugs, involve cost-sharing and most social care involves direct payments. In England, charges for prescribed pharmaceuticals have a fixed rate, and dental care in England and Wales is capped depending on treatment, and eligible populations are exempt from paying for pharmaceuticals, dental treatments, and eye tests. GPs supply care to registered patients for common conditions and are the initial contact point when a person has concerns related to their health. GPs also refer patients to specialized care usually provided by specialists in state-owned hospitals. Patients can pay to access specialist consultations privately, but this usually requires a GP referral. NHS-paid acute elective care is also available in private treatment centres in England, often in the same location as NHS hospitals. Some NHS hospitals in the UK also offer services for paying patients. For elective care in England, Scotland and Wales patients can in principle choose to go to any hospital that provides services at the same price as the NHS (see [42]).

In Poland, most of the resident population is obliged to be insured with the National Health Fund (NFZ, *Narodowy Fundusz Zdrowia*). The NFZ is a state agency which purchases from public and private providers the services included in the benefits package of insured individuals. Most of the insured population pays contributions, which are a de facto dedicated income tax, while the state budget covers the contributions for certain population groups which are exempt from paying. Individuals who are not obliged to be insured can voluntarily purchase insurance with the NFZ. Regardless of their insurance status, certain groups have the right to NFZ-covered benefits which are paid by the state. Insurance coverage is almost universal for residents of Poland. Care at the primary, outpatient specialist, and hospital level is free of charge. Inpatients of sanatoria and long-term-care institutions need to pay a room-and-board fee, and pharmaceuticals for outpatients and medical devices are subject to cost-sharing through co-payment or co-insurance. Most dental care is excluded from the insurance package. Some vulnerable and distinguished groups are entitled to access more dental services for free and are exempt from some co-payment for prescription drugs, medical devices, and treatments in sanatoria. Patients register with a primary health care physician who has a contract with the NFZ, a family doctor of their preference, or a paediatrician in the case of children. The primary health care physician helps patients with common health issues or refers them to a specialist doctor or hospital. It is possible to access without a referral specialist in the fields of dentistry, venereology, psychiatry, oncology, obstetrics, gynaecology, and until 2014 ophthalmology and dermatology. Once they are referred, patients register with any public or private specialist with a contract with the NFZ and enter a waiting list. In case patients do not want to wait they can pay out of pocket and access a public provider or a private one without a contract (see [43]).

## Methods

This article is based on the second phase of a mixed-methods study conducted at the UK level on how Polish people manage their health transnationally. The first part of the study consisted of an online survey which gathered information on access to healthcare services, and health-related and demographic characteristics between November 2019 and February 2020. Respondents (510 N) were recruited through online advertisement and email to participants in a previous questionnaire. The eligibility criteria were being over eighteen years old, being a Polish citizen or child of a Polish citizen, living or having lived in the UK, and having consulted a medical professional in the UK.

The second phase consisted of semi-structured interviews with a purposeful sample drawn from the survey respondents who identified as having a long-term health condition or caring in a non-professional capacity for someone who is chronically ill (see Table 1). This allowed us to interview participants with enduring experiences with healthcare services and to include multiple perspectives given the diversity in number and severity of the health conditions present in our sample. Of the 143 respondents invited to take part in the interviews via email, 52 accepted to be interviewed. Of these, 32 were interviewed once. During the semi-structured interviews, participants were asked to recount their and their families’ experiences with healthcare services, talk about their plans, and compare and evaluate their past experiences. Each participant was interviewed via audio call between June and August 2020 for an average of 64 minutes and was offered a small coupon as a gratitude gesture. All participants were living in England. The interviews were conducted in Polish, except for one in English following the participant’s preference. They were audio-recorded, and later translated and transcribed into English by a professional translator. The interviews and the analysis were conducted by the first author who is a Polish speaker, the son of a Polish migrant to another European country, and a migrant to the UK for ten years. The interviewees exchanged their views and experiences often referring to a shared condition as the interviewer (migrants using healthcare services in the UK), and at times their curiosity about the interviewer’s background and personal connections with Poland prompted them to further elaborate on their circumstances. However, since the interviewer has not been brought up in Poland, the participants did not give for granted the interviewer’s familiarity with navigating health services and with living in Poland and felt the need to expand upon their experiences in detail. We further detail our methodology elsewhere [18, 44]. For the analysis, thematic coding was developed and applied to all interview transcripts and coded segments were compared between interviews. The respondents were given participant information sheets before the interviews and signed consent forms. All names are pseudonyms.

**Table 1:** Participants characteristics

	%	N
**Sex**		
Female	69	22
Male	31	10
**Age groups**		
20s	6	2
30s	31	10
40s	32	11
50+	28	9
**Time in the UK**		
0–5 years	13	4
5–9 years	34	11
10–14 years	44	14
15–16 years	9	3
**Has a chronic condition**	81	26
**Cares for someone**	31	10
**Household monthly income (£)**		
Up to 1283	22	7
1283–1922	28	9
1922–2735	9	3
2735–3986	19	6
3986+	22	7
**Total sample**		32

## Results

We group our results into three sections. Firstly, we detail the recurring features which our participants referred to when evaluating UK healthcare and the differences in opinions amongst them. We find that primary and secondary care were judged strikingly differently. Secondly, we describe the reasons and kinds of specialist care accessed within the private sector. Access to specialist services privately was related to how much participants considered their needs to be attended to within the NHS, or to the extent to which it was financially advantageous in the case of services not covered by the public system. Thirdly, we explore our participants’ understanding and judgment of specialist care in their comparisons between Poland and the UK, and how their access to these services in both countries changed with migration. Participants were more familiar with the Polish healthcare system, which they judged ambiguously, while they were positive about the specialist care publicly available in the UK. The changes brought by migration allowed for greater affordability of private services when compared with the time before migration, but access to these services was contingent on the availability of time and disposable income.

### Public primary and secondary care in the UK

You can’t choose a doctor you want to see. When you want to see a doctor, the earliest appointment you can get is the following week. I never know who I’m going to see… I cannot choose who I want to see. Doctors change every three months anyway, so I always see a new person anyway. A doctor has only ten minutes for me, sharp… Within these ten minutes, they have to check my medical history on the computer, hear about the problem, often check tests’ results, make a decision about what to do next and then file all of that information on the computer… The ‘ten minute thing’ is just a nonsense. Usually, any doctor that sees me, sees me for the first time. I cannot choose to be seen by the same person. They will only ever meet me once… Still, they are supposed to find out about my problems and recommend a treatment for it… It’s simply impossible… It is sick. It is mental….

Another thing altogether is those doctors’ level of expertise… They look at you as if you were stupid; they don’t know what you’re talking about and keep telling you to eat fruit and veg five times a day, have a good quality of sleep and everything will be ok… But unfortunately, you can’t treat everything this way… Also, you can only discuss one thing during your appointment… (Dorota).

Dorota summarizes some of the most common complaints expressed by our participants who were doubtful about doctors’ competence, prescribing practices, and recommendations about diet and rest consistently with the existing literature [20–24]. Participants often recognized the appropriateness of some of these practices. Krystyna expresses a middle ground position by reflecting on the differences in approach in both countries “It’s just a completely different attitude… In Poland the approach is slightly overeager, whilst in the UK it’s the opposite…, ‘nothing’s wrong, it’s all ok’…” Other recurring issues were the waiting times to access consultation and their duration. As Dorota elucidates, participants felt constrained in their possibility to select a GP practice and being regularly cared for by a known doctor due to the capacity of GP practices to accept new patients, the extension of their catchment areas, their availability in accepting new patients, and the change of practitioners who visited them. The salience of these aspects is reflected in the fact that they were also stressed to express positive experiences with primary care.

While the satisfaction, experience and opinions around public healthcare varied, we find that the main complaints were directed towards the care at the primary level, to which Dorota refers. Participants tended to emphasize that for medical issues that seemed to be not related to common illnesses, such as cold or flu, GPs tended to be dismissive about their complaints and not investigate their health further by employing tests or referring them to specialists. While as suggested by the literature these aspects pertain to differences in clinical approaches between countries, participants understood them as a lack of promptness in treating and investigating illness. One of the main problematic aspects of primary care was its gatekeeping function towards specialized care [20, 22, 33]. When participants felt that their health needs exceeded the competence of the primary care doctors it was more difficult than in Poland to be referred to a specialist. Thus, we find that our participants’ issues with primary care were the difficulties in accessing it, the kind of care they received and the GP’s gatekeeping function.

When participants were able to access specialist care through the NHS, it was mainly commented upon positively. Maria, remarks that although she’s “only been here five years” she “had to deal with doctors here quite a bit”. She’s visited annually by a cardiologist due to her heart condition. Her husband underwent two surgeries and her father once visiting for Christmas was taken in an ambulance to a hospital. She emphasizes her experience with specialists: “They really do care… Once you get under the specialist care, they really look after you.” While once a referral to a specialist was obtained the experiences with waiting times differed amongst participants, but they did not express the strong doubts about the competence of specialists as they did for primary care doctors. “I don’t think they are better or have better training, I don’t think so… I don’t think Polish doctors aren’t good, but certainly diagnostic equipment available in the UK is much better, and that’s a big advantage…”, Maria explained, locating the source of this advantage in comparison to Poland in the better financial status of the British public system: “but that’s because the NHS here has more money…” Thus, although administered within the same public healthcare system, the care received at the primary and secondary levels is judged with a striking difference. Access to secondary care was not problematic once it was granted at the primary care level, rather it was the quality of secondary care which was contested.

### Specialist care within the private sector

We found that the most common healthcare service bought was specialist care. Odontological services were the most commonly paid-for service. Similarly to Polish people living in the UK, Spain, Norway, and Belgium where dental care requires a fee [20, 34, 35, 45], but contrary to Germany, where it is covered by work-based insurance [20], our participants whose dental care was not covered by the NHS found advantageous to access it in Poland for its comparatively lower price:

I’ll go to a dentist to have a tooth extraction; to get it done now on the NHS is unfortunately too long and private appointments are just too costly. My dentist in the UK said it was safe to wait, so I decided to get it done in Poland for the fraction of the fee. (Ilona)

Participants also paid for dental care by non-Polish professionals in the UK. For participants who did not feel proficient enough in English, Polish professionals constituted a convenient option:

I chose that because of the language. I have been to a British dentist too and the quality of service was pretty much the same. But to make things easier, so that I don’t have to take my daughter with me when I go to a dentist, I chose a Polish clinic. (Karolina)

Other kinds of specialist care were aimed at complementing primary care, supplementing specialist care already received, or accessing specialists at all through bypassing the GP gatekeeping. Emilia’s GP treated her husband for an ear infection. Not considering the treatment efficient, he complimented the primary care received within the NHS with specialist care during a visit to Poland:

He’s had problems with his ears many times and here he’s only been prescribed drops which work for two or three weeks, and then the inflammation comes back, but no one has ever done a swab test to check what the reason for the problem is. When we were in Poland, he went to see an ENT [Ear Nose and Throat] doctor privately. He took a swab sample from the ear and prescribed adequate medication, and thanks god, the problem’s gone…

Jakub has multiple medical conditions for which he has been treated in the UK and which left him not fit to work, and he recounts how he started using private services in Poland to supplement treatment which he received for free:

After I left the hospital, I had three months of physiotherapy at one of the NHS rehabilitation centres, but unfortunately after I finished, I still had to use a wheelchair. After that I had physiotherapy at home once a week, so I had one hour-long exercise session every week. We tried getting up, walking… but it just wasn’t enough. You may say that was the end of my adventure with the NHS… Obviously I continued having follow-up appointments every now and then, and things like that… But overall I was not happy with my general physical condition and that’s why I started looking into doing physiotherapy privately…

Specialists were also consulted to obtain a second opinion about their health status when there was already access to specialist care in the UK (cf. [23]):

Initially, after I moved to the UK, I often wanted to check what another doctor would say… especially when it came to my cornea erosion… before I was to have my surgery. I thought it was good, because in Poland I can get a completely different opinion from someone who is operating far away, for not a lot of money. (Marcin)

Participants also accessed specialist services that were available through GP referral only in case of suspected illness but had been directly available or routinely accessed in Poland. Ewelina sees a gynaecologist during her annual visits to Poland:

I would see a gynaecologist once a year, like I used to do when I was a girl and young woman. And this would all be national health service, not private. A gynaecology appointment would always include a complex examination, scan, etc., which is something every female friend of mine who lives in Poland has access to… It’s simply not the case here…

Access to specialists privately was related to the extent to which certain health needs were considered as addressed within the NHS. This included needs that were not, or only partially, financially covered or were considered as not covered to the extent that they had been in Poland. Specialist care was purchased also to receive reassurance about ongoing medical advice. Other needs emerged from what were considered failures of primary care and its function as the gatekeeper to specialist care.

### Specialist care between Poland and the UK

Marcin used several healthcare services, including private services in Poland and the UK (including by Polish professionals), for which he paid out of pocket and through work-provided insurance. He recounted the reluctance of his GP in referring him to a specialist who he accessed privately through insurance, which led to a diagnosis of a health issue and consequent successful surgeries.

We are happy with the healthcare system in the UK, except - and this throughout my whole stay in the UK - except of the GP filtration. I’m not talking about the GP system as a whole; I don’t have a big issue with it, but I don’t think it’s acceptable to deny a referral to someone who is seriously ill and has evidence for it, and sometimes even has private health insurance… That bit I don’t understand. Even if I didn’t have private healthcare insurance, it should be like it is in Poland, where a GP issues a referral and then someone else informs me that I have to wait a year for my appointment; that’s ok… But the fact that a GP doesn’t even want to issue a referral, I think it’s a joke….

Marcin’s comparison with the Polish system shows the extent to which access to private providers to access care speedily was a strategy familiar to participants who also recognized the ambiguity of integrating public and private care. Maria explained her view that limited resources within the NHS restricted the capacity of GPs to refer to a specialist. She was dissatisfied with the limited referrals in the UK, while expressing ambiguity about how it works in Poland, describing it as simultaneously good and “corrupted”:

But in Poland you can see a specialist privately. If you do that - if you pay - you can skip the queue and go to hospital, but here in the UK, you can’t do that… Doctors either work privately or within the national healthcare system… They don’t work in five different places… So that’s what makes it more difficult to access specialists here… That’s what’s more difficult.

While participants knew how to navigate the healthcare system in Poland through its extensive private sector because of their previous experiences, their familiarity with how to access private services and the private healthcare market in the UK varied. While Marcin expressed a good knowledge of different services depending on his circumstances, Maria’s mistaken thought that consultants either work privately or publicly shows that the two sectors were believed to be strongly separated, owing to the less developed private sector in the UK and the lack of networks and information more extensively available in Poland. Further, Marcin and Maria’s comments show that while the ways in which private and public healthcare are intertwined in Poland allow patients some control over the care received within the public system, they are also understood ambiguously. While the possibility of juggling between private and public care in Poland was understood ambiguously and as a possibility to offset delays, it was also recognized that its effect was detrimental in the case of unavailable resources. This aspect was also present in the appreciation of other features of UK public healthcare. Participants stressed that once access to specialist care was granted, it was speedier and therefore cheaper in comparison to Poland. Zuzanna, who did not hold a positive view of primary care in the UK alluded to this when expressing her satisfaction with specialist care for her nursery-aged child:

In Poland, like I said, we had to pay for every appointment, because waiting time to see a specialist on the Polish NHS was so long, that my child would probably be at least two years old before we could start achieving what we have now… We had to travel a long distance for our appointments and we had to pay for every single one of them…

Further, treatment was positively commented as free or cheaper and at times better when compared to Poland. Monika, a cancer patient who has experienced complications which did not allow her to work, reflects both on the care, medication, medical devices and aids, and the way these were only partially covered in Poland:

I get all my medication here for free. I have ‘the white card’ [i.e. medical exemption certificate] which entitles me to free prescriptions. There’s nothing like that available in Poland. You have to pay for everything; there are no concessions for things like a compression sleeve, for example. In Poland you can’t even get a prescription for it; you have to source it and buy it yourself. Here I get everything for free…

In Poland, when I was having chemotherapy and I was given a prescription for a wig, I could only choose from the cheapest wigs; only the costs of the cheapest wigs was covered; anything slightly more expensive wasn’t. The same with the breast implants - that was funded by the national health service only up to a certain value and above that it wasn’t. Things like [special] bras, for example, I had to buy myself…

Participants were aware of comparing healthcare supplied by different providers. As seen, one of the explanations for differences between the care offered in the two public systems was their respective budgets. They also recognized that they were comparing public and private providers:

First and foremost, I use NHS in the UK. In Poland I practically didn’t use public health service. I used private medical care; that’s the main difference. That’s also why healthcare in Poland seemed better - because I did everything privately. (Judyta)

Considering private care superior was partially an outcome the possibility of selecting specialists according to their status acquired by their positions within the public system:

It is hard to generalise, but overall I’d say that I trust doctors in Poland more, but then again, it’s hard to compare this, because in Poland I use private healthcare… They are true specialists as well…The gynaecologist I see is not just any gynaecologist; he’s a head physician at a hospital ward, who has a private practice as well. (Ewelina)

The perceived superiority of private healthcare also emerged by comparing private providers between the countries:

When I went privately in Poland, my doctor’s room looked like a spaceship; the amount of equipment and various gadget my dentist had in there was just astonishing, little cameras, testing equipment, etc… (Ewelina)

Participants discussed the financial affordability of healthcare services in relation to their changed circumstances and the health systems they have used. Polish people in the UK often experience ‘disruption of their occupational identities’ [46] given that they usually work in occupations below their qualification level and previous occupation in Poland [47, 48]. Concurrently, they express a sense of ‘normality’ when compared with their previous standard of living in Poland [49–51]. For many of our participants, the comparatively higher salaries meant that after migrating private services became more affordable or even within reach of their means, and the favourable pound-zloty exchange rate together with the cheaper cost of private treatment in Poland made private care in Poland more accessible.

Thus, buying services in Poland made available medical advice or complementing therapy otherwise inaccessible within the private sector in the UK, or that would have been not accessible if the person lived in Poland. As Jakub explains: “the pound goes a long way in Poland, which is why I’ve been able to afford my physiotherapy in Poland”. Even if receiving UK benefits on the ground of his disability, he explains why he sought care there:

The main reason for going to Poland was that I could afford various services there, like my physiotherapy… I couldn’t afford it in the UK. But, equally, if lived in Poland, I wouldn’t be able to afford it, because benefits in Poland are very poor.

This new purchasing capacity also allows to access services which served to reassure participants about their health and current clinical advice, accessing tests or consulting specialists to have a second opinion, as Marcin comments: “I think because an appointment with a qualified doctor in Poland is so inexpensive, it makes you want to get a second opinion, especially in case of some serious treatments.” Through comparisons with their previous experience in Poland, it emerged that in the UK participants had a sense of greater affordability akin to what research found regarding everyday consumption [50]. More specifically, they articulated this change in terms of being less dependent on personal healthcare expenditure because of greater public coverage, simultaneously being able to be reassured about the care they received through purchasing specialist consultations transnationally.

Private healthcare in the UK was out of reach for some participants and so were Polish private clinics which offered services often understood to be cheaper than general British providers. While it was often combined with family visits, travel to Poland did necessarily involve the possibility of taking time out of work and paying for travel and services accessed there. The access to Polish clinics within the UK was also subject to the availability of time and disposable income since they were concentrated in specific localities. All the participants who accessed a Polish clinic in the UK did so in London, with one also accessing a specialist in a different major English city. While participants who used private services (excluding dentists) with some regularity had a variety of familial, employment, and financial conditions, those who did not access services regularly had almost exclusively low-paid jobs or were out of work, and were more likely not to have a partner and to have dependent children. The kind of services accessed outside of public healthcare, which was mostly confined to ad hoc consultations and treatments or regular consultations with yearly frequency is shaped also by the contingency of medical access on time and money. This also means that for conditions not considered to be adequately addressed within the public system which needed regular and constant treatment or monitoring the complementation with private healthcare was not usually possible.

## Discussion

Drawing on primary data collected from Polish migrants in the UK, we have shown that migrants’ healthcare needs emerge and are attended to as migrants shift between healthcare and welfare regimes and enact exchanges across borders. Pivotal to understanding healthcare seeking is considering how the provision of care within healthcare systems intersects with these shifts and how migrants navigate the junctures between levels of care. A crucial shift happens between public healthcare systems when migrants’ access to the NHS commences and their insurance entitlements in Poland are lost. Primary care is crucial in addressing migrants’ needs as it represents the migrants’ first contact with the healthcare system in the destination country. We have shown that when these were not considered to be covered it was an outcome of the care received at the primary level and the access provided to specialist care. The extent to which the care received was sufficient was assessed in terms of the efficiency of medical practices and the migrants’ previous experience in Poland. When primary care was considered insufficient it was regarding structural elements which shaped access to it and its provision, rather than cultural features. By the same token, specialist care within the UK public system was considered positively, and was usually regarded as insufficient when not providing the continuous and regular care once available in Poland. By juxtaposing the migrants’ experiences with primary and specialist care we have shown the differences in the critical issues in migrants’ experiences with British public healthcare. Our findings confirm a level of dissatisfaction with primary care found in previous studies [20–25] while deepening and nuancing these insights through showing the differences in the evaluations and experiences that Polish migrants had with specialist services. Consistently with earlier findings [20, 21, 23, 24, 33], our participants used specialist private healthcare to access care more speedily or at all. In addition, we have also shown that these coping strategies emerged from the migrants’ encounters with both levels of care and the connections between them. Equally, shifts between different levels of state care provided migrants with the possibility of having their health needs addressed in ways which they found novel and positive in comparison to their situation before migrating.

Together with shifting between public healthcare systems, migrants also changed their position vis a vis the healthcare market. Accessing specialists privately was a familiar strategy among migrants in order to overcome the shortcomings of public healthcare. In Poland, participants entered the private sector joining public primary care with specialist private provision. This allowed for speedier specialist care while remaining within the public health system. In the UK, private specialists provided a way to access specialist care at all or additionally, or to partially complement primary care. Migrants were exposed to the private market in the UK, including providers who catered specifically for them, and the Polish healthcare market which continued to be available albeit divorced from state-funded care. Migrants accessed these services from their position within the labour and welfare regimes in the UK and according to it enacted exchanges between borders. This was seen as a possible enhancement of possibilities to purchase healthcare, and shaped the emergence of the need to be reassured about their current treatments fulfilled by obtaining multiple medical evaluations. In the case of healthcare access, the changing occupational conditions [46–48] and the sense of normality [49–51] highlighted in previous studies about Polish migrants in the UK, are reflected in a sense of greater accessibility to publicly provided services *and* increased affordability of private care. By situating migrants’ healthcare-seeking behaviours within their changed circumstances we have also shown that specialist care responds to needs emerging because of these changes that also make possible their fulfilment. Also, access to private specialists remained contingent on the possibility to move and purchase these services. Thus, with changing circumstances new needs and possibilities to access specialist care emerged at the same time as new vulnerabilities, which together shaped the kinds of service used and the migrants’ overall healthcare experiences. Jakub’s description of economic calculus adequately characterized an element of healthcare seeking and the easiness of access to which he refers portrays many experiences with the NHS. In particular, this often characterized the access to dental care, which is usually subject to out-of-pocket payments and non-dependent on connections with primary care. Notably, this is consistent with other studies which point to the prominence of accessing dental care when it is not free in the country of residence [20, 34, 35, 45], and to the lesser conspicuousness of this behaviour when such care is freely available [20]. However, access to paid services at large also emerged as a strategy to overcome difficulties in receiving care deemed needed both at the primary and specialist level and in addition to the services already freely available. By focusing on junctures between primary and specialist care we have highlighted how the concurrent fulfilment and emergence of healthcare requires and results in customised service use within a transnational migratory context.

Migration also meant shifts between approaches to patient care and in the patient-doctor relationship. Our participants had different and often contrasting opinions about the preference for the Polish approach to care. When preferences were expressed usually focused on features of primary care highlighted in the literature [20–25] which were considered lacking in the UK such as shortness of consultations and long waiting times, and lack of competence about treatments and illnesses. Conversely, specialist doctors working in the NHS were not so much believed to be inferior to their counterparts in Poland. Dorota’s remarks about Polish doctors need to be considered within the direct access provided by specialist private care, and the availability of time this allowed. Further, makers of higher standards associated with private care, such as more cutting-edge equipment and technological devices, are used to compare private providers across borders. The clear comprehension of accessing and comparing private and public services shows that the critiques of British healthcare are better understood as critiques of its primary care and its junctures with private care, rather than a general affective attachment to Polish medical culture. Karolina’s remarks point to the fact that while the difference in approaches exists, they are actively navigated by migrants who can be satisfied with the care they receive within the public sector. Rather than a general cultural preference detached from other aspects of care, preference for “Polish” approaches to care encompasses structural and organizational aspects that when considered to be absent are chiefly located at the primary care level and its “English approach” [20], and as in the case of Dorota are found within private providers catering specifically for these needs. As we have shown, rather than their nationality per se, the preference for Polish private specialists is marked by the possibility and affordability of choosing them, and by the needs that they can fulfil given the care migrants receive within the public sector. As for the Mexican migrants studied by Horton [14], stated differences in medical cultures need to be situated within the shifting access to public and private services and the comparison between them given migrants’ changing positionalities. However, we find that equally important in these comparisons are the newly found affordability of public services and the contrasting features between home and host country private sectors to which migrants were not unaccustomed. The way in which such services are accessed and the kind of care that migrants obtain, we have argued, has its roots in how needs emerge and are fulfilled at the junctures *within* and *between* healthcare systems and levels of care.

## Study strengths and limitations

The main strength of this study is its relevance for understanding access to healthcare for migrants in the UK. Polish people are the largest migrant group in the country and this study contributes to deepening our comprehension of their interactions with services at different levels of the healthcare system. The main limitations of this study concern sampling. We interviewed more women (69%) than men and all participants were residing in England. The great majority of participants (78%) had been living in the U.K. between 5 and 14 years, while the rest have resided there less than 4 or more than 15 years. It will be important to assess the healthcare practices of Polish people in the rest of the UK and residents for a more extended period of time.

## Conclusion

Focusing on junctures highlights at once how Polish migrants understand and seek care, and in which ways their health seeking behaviours are shaped by the systemic features of the systems they navigate. Moreover, it allows the specification of how migrants link service provision between sectors. Within national frameworks, the mixture between private and public services permits a certain space of manoeuvre to obtain care while fragmenting healthcare and establishing inequality in access [52, 53]. Within a transnational framework, migrants contribute to the development of a private market in their country of origin [15, 18] where they experience changing medical practices [17]. We have shown that the integration of public services is largely dependent upon transnational practices, rather than on nationally available services. At once, migrants join with their practices systems which are not integrated, and their access is limited by the constraints implied in accessing paid services in Poland. This shapes transnational healthcare practices as relating mostly to routine and ad-hoc access to healthcare. Since they shift between sectors in multiple ways, including coming back to the public healthcare sector for the same health issues [18], these practices impact not only the wellbeing of migrants and the development of the private market but also the public health provision of services. A focus on junctures as the systemic features of healthcare systems by which migrants understand and seek care can provide insights into how migrants participate in the transformation of health services.

## Data Availability

The datasets generated and analysed during the current study are not publicly available due to their confidential nature, but are available from the corresponding author on reasonable request.

## List of abbreviations

GP: General Practitioners
NFZ: National Health Fund, *Narodowy Fundusz Zdrowia* (Poland)
NHS: National Health Service (UK)

## References

[1] ONS. Population of the UK by country of birth and nationality: year ending June 2020. 2020.

[2] Phillimore J, Bradby H, Knecht M, Padilla B, Pemberton S (2019). Bricolage as conceptual tool for understanding access to healthcare in superdiverse populations. Soc Theory Health.

[3] Freitas Cd, Mendes Á (2013). A resiliência da saúde migrante: itinerários terapêuticos plurais e transnacionais. REMHU.

[4] Glinos IA, Baeten R, Helble M, Maarse H (2010). A typology of cross-border patient mobility. Health place.

[5] Migge B, Gilmartin M (2011). Migrants and healthcare: investigating patient mobility among migrants in Ireland. Health Place.

[6] Mathijsen A, Mathijsen FP (2020). Diasporic medical tourism: a scoping review of quantitative and qualitative evidence. Global Health.

[7] Bolton S, Skountridaki L (2017). The medical tourist and a political economy of care. Antipode.

[8] Bergmann S (2011). Fertility Tourism: Circumventive Routes That Enable Access to Reproductive Technologies and Substances. Signs (Chic).

[9] Kangas B (2011). Complicating Common Ideas about Medical Tourism: Gender, Class, and Globality in Yemenis’ International Medical Travel. Signs (Chic).

[10] Horsfall D. Medical tourism from the UK to Poland: how the market masks migration. J Ethn Migr Stud. 2019:1–19.

[11] Villa-Torres L, Gonzalez-Vazquez T, Fleming PJ, González-González EL, Infante-Xibille C, Chavez R (2017). Transnationalism and health: a systematic literature review on the use of transnationalism in the study of the health practices and behaviors of migrants. SSM.

[12] Crocker RM (2021). The Impact of Binational Barriers to Medical Care on the Care-Seeking Practices of Mexican Immigrants. Qual Health Res.

[13] Horton S, Cole S (2011). Medical returns: seeking health care in Mexico. SSM.

[14] Horton SB (2013). Medical returns as class transformation: situating migrants’ medical returns within a framework of transnationalism. Med Anthropol.

[15] Ormond M, Thomas F, Gideon J (2013). Harnessing “diasporic” medical mobilities. Migration, Health and Inequality.

[16] Stan S (2015). Transnational healthcare practices of Romanian migrants in Ireland: Inequalities of access and the privatisation of healthcare services in Europe. SSM.

[17] González-Vázquez T, Infante-Xibille C, Villa-Torres L, Reyes-Morales H, Pelcastre-Villafuerte BE (2020). Collateral effect of transnational migration: the transformation of medical habitus. Salud Publica Mex.

[18] Troccoli G, Moreh C, McGhee D, Vlachantoni A (2022). Transnational healthcare as process: multiplicity and directionality in the engagements with healthcare among Polish migrants in the UK. J Ethn Migr Stud.

[19] Krause K (2008). Transnational Therapy Networks among Ghanaians in London. J Ethn Migr Stud.

[20] Main I (2018). Lepsze światy medyczne?.

[21] Osipovič D (2013). ‘if I get ill, It’s onto the plane, and off to Poland.’use of health care services by polish migrants in London. Cent East Eur Migr Rev.

[22] Madden H, Harris J, Blickem C, Harrison R, Timpson H (2017). “Always paracetamol, they give them paracetamol for everything”: a qualitative study examining Eastern European migrants’ experiences of the UK health service. BMC Health Serv Res.

[23] Sime D (2014). ‘I think that Polish doctors are better’: Newly arrived migrant children and their parents׳ experiences and views of health services in Scotland. Health Place.

[24] Bell S, Edelstein M, Zatoński M, Ramsay M, Mounier-Jack S (2019). ‘I don’t think anybody explained to me how it works’: qualitative study exploring vaccination and primary health service access and uptake amongst Polish and Romanian communities in England. BMJ Open.

[25] Goodwin R, Polek E, Goodwin K (2012). Perceived Changes in Health and Interactions With “the Paracetamol Force” A Multimethod Study. J Mix Methods Res.

[26] Gorman D, Porteous L (2018). Influences on Polish migrants’ breast screening uptake in Lothian. Scotl Public Health (Fairfax).

[27] Jackowska M, von Wagner C, Wardle J, Juszczyk D, Luszczynska A, Waller J (2012). Cervical screening among migrant women: a qualitative study of Polish, Slovak and Romanian women in London, UK. J Fam Plann Reprod Health Care.

[28] Bielecki K, Kirolos A, Willocks L, Pollock K, Gorman D (2019). Low uptake of nasal influenza vaccine in Polish and other ethnic minority children in Edinburgh. Scotl Vaccine.

[29] Pollock K, Tait B, Tait J, Bielecki K, Kirolos A, Willocks L (2019). Evidence of decreased HPV vaccine acceptance in Polish communities within Scotland. Vaccine.

[30] Gorman DR, Bielecki K, Willocks L, Pollock K, Munshaw J, Larson H. Information sources and attitudes of Polish migrants accepting or declining vaccination in Scotland. Eur J Public Health. 2019;29(Supplement_4).

[31] Crowther S, Lau A (2019). Migrant polish women overcoming communication challenges in Scottish maternity services: A qualitative descriptive study. Midwifery.

[32] Henderson J, Carson C, Jayaweera H, Alderdice F, Redshaw M (2018). Recency of migration, region of origin and women’s experience of maternity care in England: Evidence from a large cross-sectional survey. Midwifery.

[33] Smoleń A. Problemy zdrowotne polskich emigrantów poakcesyjnych. Implikacje dla systemów opieki zdrowotnej. Problemy Zarządzania. 2013(1/2013 (41) t. 2):227 – 39.

[34] Mathijsen A (2019). Home, sweet home? Understanding diasporic medical tourism behaviour. Exploratory research of Polish immigrants in Belgium. Tour Manag.

[35] Struzik J, Bell J, Pustułka P, Ślusarczyk M. Handling ambivalence: transnational health practices and migrant evaluations of health services. In Magdalena Ś, Paulina P & Justyna S editors. Contemporary migrant families: actors and issues. Newcastle upon Tyne: Cambridge Scholars Publishing: 2018. p. 48–73.

[36] Tiilikainen M, Koehn PH (2011). Transforming the Boundaries of Health Care: Insights from Somali Migrants. Med Anthropol.

[37] Calvasina P, Muntaner C, Quiñonez C (2015). Transnational dental care among Canadian immigrants. Community Dent Oral Epidemiol.

[38] Moreh C, McGhee D, Vlachantoni A. EU migrants’ attitudes to UK healthcare. ESRC Centre for Population Change Briefing Paper 41. 2018.

[39] Gideon J (2011). Exploring migrants’ health seeking strategies: the case of Latin American migrants in London. Int J Migr Health Soc Care.

[40] Guma T (2018). Exploring Potentialities of (Health) Care in Glasgow and Beyond: Negotiations of Social Security Among Czech-and Slovak-Speaking Migrants. Cent East Eur Migr Rev.

[41] Thomas F (2010). Transnational health and treatment networks: meaning, value and place in health seeking amongst southern African migrants in London. Health Place.

[42] Cylus JRE, Findley L, Longley M, O’Neill C, Steel D (2015). United Kingdom: Health system review. Health Syst Transition.

[43] Sowada CSA, Kowalska-Bobko I, Badora-Musiał K, Bochenek T, Domagała A, Dubas-Jakóbczyk KKE, Mrożek-Gąsiorowska M, Sitko S, Szetela A, Szetela P, Tambor M (2019). Więckowska B Z-JM, van Ginneken E. Poland: Health system review. Rep No.

[44] Troccoli G, Moreh C, McGhee D, Vlachantoni A (2022). Diagnostic testing: therapeutic mobilities, social fields, and medical encounters in the transnational healthcare practices of Polish migrants in the UK. J Migr Health.

[45] Czapka EA, Sagbakken M (2016). “Where to find those doctors?” A qualitative study on barriers and facilitators in access to and utilization of health care services by Polish migrants in Norway. BMC Health Serv Res.

[46] McGhee D, Travena P, Heath S (2015). Social Relationships and Relationships in Context: Post-Accession Poles in Southampton: Post-Accession Poles in Southampton. Popul Space Place.

[47] Drinkwater S, Eade J, Garapich M (2009). Poles apart? EU enlargement and the labour market outcomes of immigrants in the United Kingdom. Int Migr.

[48] Lopez Rodriguez M (2010). Migration and a quest for ‘normalcy’. Polish migrant mothers and the capitalization of meritocratic opportunities in the UK. Soc Identities.

[49] Galasińska A, Kozłowska O. Discourses of a ‘Normal Life’ among Post-accession Migrants from Poland to Britain. In: Burrell K, editor. Polish Migration to the UK in the’new’European Union: After 2004. Routledge; 2009. pp. 87–105.

[50] McGhee D, Heath S, Trevena P (2012). Dignity, happiness and being able to live a ‘normal life’ in the UK – an examination of post-accession Polish migrants’ transnational autobiographical fields. Soc Identities.

[51] White A (2017). Polish families and migration since EU accession.

[52] Węgrzynowska M (2021). Private Services and the Fragmentation of Maternity Care in Poland. Med Anthropol.

[53] Stan S, Toma V-V (2019). Accumulation by Dispossession and Public–Private Biomedical Pluralism in Romanian Health Care. Med Anthropol.

